# Supplementary material for: A Comprehensive Integrated Genetic Map of the Complete Karyotype of Solea senegalensis (Kaup 1858)
Source: Genes (Basel). 2020 Dec 31;12(1):49. doi: 10.3390/genes12010049 (PMC7824234; doi:10.3390/genes12010049)
Supplement: Supplementary file 1 [file genes-12-00049-s001.zip › Table S1.docx]

**Table S1.** BAC-FISH, sequencing and reference data of all BAC clones used. MT: Metacentric chromosome; SMT: Submetacentric chromosome; STL: Subtelocentric chromosome; TL: Telomeric chromosome; C: Centromeric location; Pc: Pericentromeric location; Sc: Subcentromeric location; I: Interstitial location; Stl: Subtelomeric location. Tl: Telomeric location. Underlined locations refer to the main signal of a multiple signal hybridization.

| **BAC clone** | **Size of all contigs** | **Genome coverage^a^** | **Chromosome-location** | **Annotated genes** | **References** | **NCBI Accession Number** |
| --- | --- | --- | --- | --- | --- | --- |
| 1C2 | 44432 | 0,007 | MT1-Tl | *Neurobeachin (****nbea****)* | García-Angulo et al. 2018 | MW199153.1 |
| 2K18 | 64715 | 0,011 | Dispersed | *Cytochrome c oxidase protein 20 (****cox20****), Luteinizing hormone beta subunit 4 (****lhb****), Nuclear matrix constituent protein 2 (****nmcp2****), Reticulon 4 (****rtn4****), Transmembrane 9 superfamily member 2 (****tm9sf2****), Ubiquitin conjugating enzyme (****ube2g1****), LINE-1 type transposase domain-containing protein 1 (****L1td1****)* | Portela-Bens et al. 2017 | AC270124.1 |
| 3C15 | 79261 | 0,013 | SMT4-Stl | *Cytochrome c (****cycsb****), Oxysterol-binding protein (****osbpl6****), Dual 3',5'-cyclic-AMP and -GMP phosphodiesterase 11a (****pde11a****)* | García et al. 2019 | AC278070.1 |
| 3F15^c^ | 1909958^b^ | 0,312 | MT2-I  TL14-Stl  TL17-Tl | *Leucine rich repeat (****lrit1****), RPE Retinal G protein coupled receptor (****rgr****), Probable vesicular acetylcholine transporter (****slc18a3a****)* | Present work | MW199152.1 |
| 3N10 | 54814 | 0,009 | TL11-Stl | *Solute carrier family 25 member 47-A (****slc25a47a****), Vertnin (****vrtn****), Mitochondrial basic amino acids transporter (****slc25a29****)* | Present work | AC278092.1 |
| 4D15 | 75166 | 0,012 | MT2-Tl | *Protein phosphatase methylesterase 1* *(****ppme1****), Ring finger protein 150* *(****rnf150****), Inositol polyphosphate 4-phosphatase type II* *(****inpp4b****), High mobility group protein 2* *(****hmgb2****), Disks large homolog 4* *(****dlg4****)* | García-Angulo et al. 2019 | AC278120.1 |
| 4E10 | 392275^h^ | 0,064 | TL11-I | *Monofunctional c1-tetrahydrofolate synthase, mitocondrial (****mthfd1l****),* *Syntaxin-11 (****stx11****)* | García et al. 2019 | AC278061.1 |
| 4F12 | 68549 | 0,011 | TL15-Sc | *Golgi snap receptor complex member 2 (****gosr2****), G patch domain-containing protein 8 (****gpatch8****), Protein wnt-9b (****wnt9b****), ADP-ribosylation factor 1 (****arf1****)* | García et al. 2019 | AC278102.1 |
| 4M14 | 283462 | 0,046 | TL20-I | *Osteoclast stimulatory transmembrane protein* *(****ocstamp****), Scratch family transcriptional repressor 2* *(****scrt2****), Solute carrier family 13 member 3 (****slc13a3****), Transcription factor 15* *(****tcf15****), Thyrotropin releasing hormone receptor 3* *(****trhr3****)* | García-Angulo et al. 2020 | AC278088.1 |
| 4N21 | 57915 | 0,009 | TL13-I | *Lysine export transcriptional regulatory protein LysG (****lysg****), Peptidoglycan-recognition protein 2 (****pglyrp2****), Mucolipin 1 (****mcoln1****)* | Ponce et al. 2011 | FR870080.1 |
| 5K5 | 704705^e^ | 0,115 | MT1-Tl | *Tropomyosin alpha-4 chain (****tpm4****), Krueppel-like factor 2 (****klf2****), Epidermal growth factor receptor substrate like 1 (****eps15l1****), Ras-related protein Rab-8A (****rab8a****), Calcium and integrin-binding family member 3 (****cib3****), Excitatory amino acid transporter 1 (****slc1a3****), 2x Histone H2A (****H2a****), 2x Histone H3 (****H3****), 3x Histone H4 (****H4****), 3x Histone H2B (****H2b****), Histone H1 (****H1****), Calreticulin (****calr****), Retinal homeobox protein Rx2 (****rx2****), Tropomyosin alpha-1 chain (****tpm1****), AP-1 complex subunit mu-1 (****ap1m1****)* | Merlo et al. 2017 | AC275288.1 |
| 6P22 | 54029 | 0,009 | MT2-Stl | *Leydig cell tumor 10 kDa protein homolog (****c19orf53****), Coiled-coil and C2 domain-containing protein 1a (****cc2d1a****), Centriolar coiled-coil protein of 110 kDa (****cpp110****), DDB1- and CUL4-associated factor 15 (****dcaf15****), Guanine nucleotide-binding protein G(o) subunit alpha (****gnao1****), Guanine nucleotide-binding protein G(I)/G(S)/G(O) subunit gamma-10 (****gng10****), Far upstream element-binding protein 2 (****khsrp****), Transcription factor MafG (****mafg****), Dual specificity mitogen-activated protein kinase 5 (****map2k5****), Methylthioribose-1-phosphate isomerase (****mri1****), Nanos homolog 3 (****nanos 3****), Neuropeptide B (****npb****), Ethanolamine-phosphate cytidylyltransferase (****pcyt2****), MHC class II regulatory factor RFX1 (****rfx1****), Regulator of G-protein signaling 5 (****rgs5****), Sodium-independent sulfate anion transporter (****slc26a11****), N-sulphoglucosamine sulphohydrolase (****sgsh****), NAD-dependent protein deacetylase sirtuin-7 (****sirt7****), SKI family transcriptional corepressor 1 homolog-B (****skor1b****), Calcium-binding mitochondrial carrier protein SCaMC-1 (****slc25a24****), Afadin- and alpha-actinin-binding protein (****ssx2ip****), Uromodulin (****umod****)* | Portela-Bens et al. 2017 | AC270104.1 |
| 7H22 | 112198 | 0,018 | STL7-Sc | *ADP-ribosylation factor-like protein 14 (****arl14ep****), Follicle-stimulating hormone beta subunit (****fshβ****), Potassium voltage-gated channel subfamily A member 4 (****kcna4****), MAP kinase-activating death domain protein (****madd****), Oxysterols receptor LXR-alpha (****nr1h3****)* | Portela-Bens et al. 2017 | MW199154.1 |
| 8A23 | 100967 | 0,016 | SMT4-Sc | *tRNA-specific adenosine deaminase-like protein 3 (****adat3****), Small membrane A-kinase anchor protein (****akap****), Alpha-ketoglutarate-dependent dioxygenase alkB homolog 6 (****alkbh6****), Peptidyl-prolyl cis-trans isomerase FKBP7 (****fkbp7****), Glutaminase kidney isoform, mitocondrial (****gls****), 3-hydroxyisobutyryl-CoA hydrolase, mitocondrial (****hibch****), Myostatin (****mstn****), ORM1-like protein 1 (****ormdl1****), Probable tRNA N6-adenosine threonylcarbamoyltransferase, mitochondrial (****osgepl1****), Post-meiotic segregation 1 protein homolog 1 (****pms1****)* | García et al. 2019 | AC278106.1 |
| 8O7 | 176831 | 0,029 | STL7-Pc  TL13-Stl | *Calcitonin gene-related peptide 2 (****calcb****), Ethanolamine kinase21 (****etnk2****), Protein inscuteable homolog (****insc****), Rhombotin-1 (****lmo1****), Proteasome subunit alpha type-1 (****scl1****), Ras-related and estrogen-regulated growth inhibitor-like protein (****rergl****), Protein resistant to inhibitor of cholinesterase 3 (****ric3****), SRY-related HMG-box (****sox 6****)* | Potela-Bens et al. 2017 | AC270096.1 |
| 9E8 | 1909958^b^ | 0,312 | MT2-Stl  TL10-Sc | *Nuclear receptor subfamily 1 group D member 2* *(****nr1d2****), Thyroid hormone receptor beta* *(****trβ****), E2 ubiquitin-conjugating enzyme E2* *(****ube2e2****)* | García-Cegarra et al. 2013 | MW199155.1 |
| 9J4 | 140126 | 0,023 | MT3-Stl | *Uncharacterized protein C14orf28 (****C14orf28****), Ankyrin repeat domain-containing protein 63 (****ankrd63****), Coiled-coil domain-containing protein 28B (****ccdc28b****), Protein FAM179B (****fam179b****), KH domain-containing, RNA-binding, signal transduction-associated protein 1 (****khdrbs1****), Kelch-like protein 28 (****klhl28****), c-1-tetrahydrofolate synthase, cytoplasmic (****mthfd1****), Serine/threonine-protein kinase PAK 6 (****pak6****), 1-phosphatidylinositol 4,5-bisphosphate phosphodiesterase beta-2 (****plcb2****), Histone-binding protein RBBP4 (****rbbp4****), Transmembrane protein 39B (****tmem39b****), coiled-coil domain-containing protein 9B (****ccdc9b****)* | García et al. 2019 | AC278104.1 |
| 9N8 | 51170 | 0,008 | TL16-Stl | *Mx type 2 (****mx2****), Semaphorin 7A (****sema7a****)* | García-Cegarra et al. 2013 | AC278057.1 |
| 10K23 | 704705^e^ | 0,115 | MT1-Stl | *Rho GTPase-activating protein 21-A (****arhgap21****), Teleost multiple tissue opsin b (****tmtopsb****), Opsin-3 (****opn3****), Calreticulin (****calr****), Otospiralin (****otos****), Epidermal growth factor receptor substrate 15-like 1 (****eps15l1****), Apolipoprotein D (****apod****), Actinodin 1 (****and1****)* | Portela-Bens et al. 2017 | AC278118.1 |
| 10L10 | 704705^e^ | 0,115 | MT1-Stl | *Krueppel-like factor 2 (****klf2****), Epidermal growth factor receptor substrate 15-like 1 (****eps15l1****), Retinal homeobox protein Rx2 (****rx2****), Calreticulin (****calr****)* | García-Angulo et al. 2018 | AC278108.1 |
| 11O20 | 256445^f^ | 0,042 | STL6-Tl | *Arrestin domain-containing protein 3 (****arrdc3****), Aquaporin-3 (****aqp3****), Nucleolar protein 6 (****nol6****)* | Portela-Bens et al. 2017 | AC278084.1 |
| 12D22 | 49915 | 0,008 | MT1-Sc | *3x Histone H2A (****H2a****), 3x Histone H3 (****H3****), 2x Histone H4 (****H4****), 5x Histone H2B (****H2b****), 2x Histone H1 (****H1****), Ankyrin repeat domain-containing protein 45 (****ankrd45****), Transmembrane protein 70, mitochondrial (****tmem70****)* | Merlo et al. 2017 | AC275286.1 |
| 12D24 | 183576 | 0,030 | SMT4-I | *Calsequestrin 2a (****casq 2a****), Dopey2 (****dop1b****), Immunoglobulin superfamily member 3 (****igsf3****), Potassium voltage-gated channel subfamily E member 2 (****kcne2****), MORC family CW-type zinc finger protein 3 (****morc3****), Helix-loop-helix protein 2 (****nhlh2****), NAD(P)H dehydrogenase, quinone 1 (****nqo1****), Receptor-type tyrosine-protein phosphatase-like N (****ptprn****), Ribulose-phosphate 3-epimerase (****rpe****), Sodium/myo-inositol cotransporter (****slc5a3****), Vang-like 1 (****vangl1****), Atypical chemokine receptor 3 (****ackr3****)* | Arias-Pérez et al. 2018 | AC278101.1 |
| 12K16 | 20330 | 0,003 | TL19-Sc | *Interferon-induced GTP-binding protein Mx (****mx****)* | García-Cegarra et al. 2013 | AC278051.1 |
| 12N15 | 162898 | 0,027 | SMT4-Sc | *Ankyrin repeat domain-containing protein 10 (****ankrd10****), Sodium/potassium-transporting ATPase subunit beta-233 (****atnb233****), Cyclic nucleotide-gated channel cone photoreceptor subunit alpha (****cnga****), Vasa Probable ATP-dependent RNA helicase (****vasa****), Inactive dipeptidyl peptidase 10 (****dpp10****), Glycerol-3-phosphate dehydrogenase, mitocondrial (****gpd2****), Heat shock factor 2-binding protein (****hsf2bp****), Nuclear receptor subfamily 4 group A member 2 (****nr4a2****), Trace amine-associated receptor 13c (****taar13c****), Tubulin alpha-1C chain (****tuba1c****), Ubiquitin-protein ligase E3A (****ube3a****), Coiled-coil domain-containing protein 14 (****ccdc14****)* | Portela-Bens et al. 2017 | AC270101.1 |
| 13E1 | 1909958^b^ | 0,312 | TL12-I | *DEAH-box helicase 16* *(****dhx8****), ETS variant transcription factor 4* *(****etv4****), Thyroid hormone receptor alpha b* *(****thrαb****)* | García-Cegarra et al. 2013 | MW199156.1 |
| 13F2 | 26326 | 0,004 | TL12-Sc | *No genes annotated* | García et al. 2019 | AC278062.1 |
| 13F4 | 88852 | 0,015 | TL19-Sc | *No genes annotated* | García et al. 2019 | AC278086.1 |
| 13G1 | 15554 | 0,003 | MT1-Stl | *WW domain-containing adapter protein with coiled-coil (****wac****)* | García-Angulo et al. 2018 | AC278085.1 |
| 15I19 | 203915 | 0,033 | TL10-Sc | *Death domain-associated protein 6 (****daxx****), Flotillin 1 (****flot1****), Carboxy-terminal kinesin 2 (****kifc1****), Transcription factor 19 (****tf19****), Tubulin beta chain (****tubb****), Zinc finger and BTB domain-containing protein 22 (****zbtb22****), Zinc finger protein 384 (****znf384****)* | García et al. 2019 | AC278066.1 |
| 16E16 | 272508^g^ | 0,045 | MT1-Sc  STL6-Sc  TL15- I | *Doublesex and mab-3-related transcription factor 2 (****dmrt2****), Doublesex and mab-3-related transcription factor 3 (****dmrt3****)* | Portela-Bens et al. 2017 | AC270103.1 |
| 19H9 | 72971 | 0,012 | STL7-I | *Cytocrome P450 aromatase (****cyp19a1a****), Dmx-like protein 2 (****dmxl2****), Gliomedin (****gldn****)* | Portela-Bens et al. 2017 | AC270100.1 |
| 19J21 | 319461 | 0,052 | MT2-Sc  TL15-I | *Carbonyl reductase family member 4 (****cbr4****), Coiled-coil domain-containing protein 149-B (****ccdc149b****), Putative pre-mRNA-splicing factor ATP-dependent RNA helicase DHX15 (****dhx15****), F-box/LRR-repeat protein 5 (****fbxl5****), Mediator of RNA polymerase II transcription subunit 9 (****med9****), Paladin (****palld****), Dexamethasone-induced Ras-related protein 1 (****rasd1****), E3 ubiquitin-protein ligase (****sh3rf1****), SRY-related HMG-box 9 (****sox9****), Somatostatin receptor type 2M (****sstr2****), Tripartite motif-containing protein 16 (****trim16****), Ubiquitin-specific-protease-3 (****usp3l****), Serine/threonine-protein kinase Nek1 (****nek1****), Coiled coil domain containing protein 149-B (****ccdc149b****), Serine/threonine-protein kinase WNK1 (****wnk1****)* | Portela-Bens et al. 2017 | MW199157.1 |
| 19K18^c^ | 1909958^b^ | 0,312 | STL9-Sc  TL16-I  TL17-I | *CUGBP Elav-like family member 4 (****celf4****), Endothelial cell selective adhesion molecule (****esam****), Fasciculation and elongation protein zeta 1 (****fez1****), Immunoglobulin superfamily member 9B (****igsf9b****), Cilia and flagella associated protein 58 (****cfap58****)* | Present work | MW199158.1 |
| 19L16^c^ | 1909958^b^ | 0,312 | MT2-Stl  TL13-Tl  TL-Sc | *Chromosome 1 open reading frame 194 (****c1orf194****), Protein FAM107B (****fam107b****), UPF0577 protein KIAA1324 (****kiaa1324****), Structural maintenance of chromosomes protein 5 (****smc5****)* | Present work | MW199159.1 |
| 20D18 | 256445^f^ | 0,042 | STL6-Tl | *Aquaporin-3 (****aqp3****), Drebrin-like protein A (****dbnl-a****), Histone deacetylase 11 (****hdac11****), Nucleolar protein 6 (****nol6****), Rho-related BTB domain-containing protein 2 (****rhobtb2****), WD repeat-containing protein 54 (****wdr54****), Arrestin domain-containing protein 3 (****arrdc3****), Zinc finger BED domain-containing protein 1 (****zbed1****)* | Portela-Bens et al. 2017 | MW199160.1 |
| 21O23 | 54341 | 0,009 | MT2-Stl  TL14-Tl | *Doublesex and mab-3-related transcription factor a1 (****dmrta1****), Intestinal fatty acid-binding protein (****fabp2****), Pituitary adenylate cyclase-activating polypeptide type I receptor (****adcyap1r1****)* | Portela-Bens et al. 2017 | AC270102.1 |
| 22C2 | 1909958^b^ | 0,312 | TL15-Tl | *BR serine/threonine kinase 2 (****brsk2****), Dual specificity protein phosphatase (****dusp3****), Prolactin (****prl****), Thyroid hormone receptor alpha A (****thrαa****)* | García-Cegarra et al. 2013 | MW199161.1 |
| 25P16^c^ | 1909958^b^ | 0,312 | TL17-Tl  TL21-Stl | *Eukaryotic translation initiation factor 3 subunit E-A (****eif3ea****), ER membrane protein complex subunit 2 (****emc2****), Microtubule-associated serine/threonine-protein kinase 3* *(****mast3****), Transmembrane protein 74 (****tmem74****), Thyrotropin-releasing hormone receptor 1a (****trhr1a****)* | García-Angulo et al. 2020 | MW199151.1 |
| 29D4 | 198050 | 0,032 | TL13-Sc | *Sorbin and SH3 domain-containing 2a* *(****sorbs2a****), Toll-like receptor 3* *(****tlr3****), Cytochrome P450 4V2 (****cyp4v2****), Melatonin receptor type 1A-A* *(****mtnr1aa****)* | García-Angulo et al. 2019 | AC278089.1 |
| 30H22 | 190392 | 0,031 | TL21-Stl | *Anti-Mullerian hormone (****amh****), Histone-lysine N-methyltransferase, H3 lysine-79 specific (****dot1l****), RNA polymerase II elongation factor ELL (****ell****), Peptidyl-prolyl cis-trans isomerase FKBP8 (****fkbp8****), Ornithine decarboxylase antizyme 1 (****oaz1****), Inactive tyrosine-protein kinase PRAG1* *(****prag1****), Single-stranded DNA-binding protein 3 (****ssbp3****), C2 calcium-dependent domain-containing protein 4C (****c2cd4c****)* | Portela-Bens et al. 2017 | AC270099.1 |
| 30J4 | 372766 | 0,061 | SMT4-Sc  TL12-Tl | *Protocadherin-8* *(****pcdh8****), Endothelin receptor type B* *(****ednrb****), Component of oligomeric golgi complex 3 (****cog3****), E3 ubiquitin-protein ligase Midline-1* *(****mid1****), Rho GTPase-activating protein 6* *(****arhgap6****), FERM and PDZ domain-containing protein 4* *(****frmpd4****), Toll-like receptor 7 (****tlr7****), Toll-like receptor 8*  *(****tlr8****), Thymosin beta* *(****tyb12****), Epidermal* *growth factor-like protein 6* *(****egfl6****), NLR family, CARD domain-containing 3 (****nlrc3****), Glucagon receptor* *(****gcgr****), WD repeat-containing protein 90* *(****wdr90****), Mitochondrial Rho GTPase 2* *(****rhot2****), Histone H1.0* *(****h1f0****), Rhomboid-related protein 1* *(****rhbdl1****), WD repeat-containing protein 24* *(****wdr24****), Ankyrin repeat and SAM domain-containing protein 3* *(****anks3****), UPF0488 protein C8orf33 (****upfpcc8orf33****), Histone H3* *(****h3.3****), G-protein coupled estrogen receptor 1 (****gper1****)* | Merlo et al. 2017 | AC275287.1 |
| 30P17 | 85259 | 0,014 | TL16-I | *Target of Nesh-SH3 (****abi3bp****), Collagen alpha-1(VIII) chain (****col8a1****), Transcription iniciation factor (****tfg****)* | García et al. 2019 | AC278054.1 |
| 31A1 | 76388 | 0,012 | STL8-Tl | *Protein-unc-45 homolog B (****unc45b****), Kinesin-like protein KIF2A (****kif2a****), Probable dimethyladenosine transferase (****dimt1****), Centromere protein H (****cenph****), Betaine--homocysteine S-methyltransferase 1 (****bhmt****), Junction-mediating and -regulatory protein (****jmy****)* | García et al. 2019 | AC278116.1 |
| 31A2 | 70097 | 0,011 | STL8-Stl | *Semaphorin-4B (****sema4b****), Ras and EF-hand domain-containing protein (****rasef****), Peptidyl-prolyl cis-trans isomerase (****fkbp8****)* | García et al. 2019 | AC278068.1 |
| 31B1 | 10301 | 0,002 | Dispersed | *No genes annotated* | Present work | AC278091.1 |
| 31C1 | 169459 | 0,028 | TL19-Tl | *Recombination signal-binding protein for immunoglobulin kappa J region-like* *(****rbpjl****), Tripartite motif-containing* *protein 16 (****trim16****), Matrilin-4* *(****matn4****), Odorant receptor 134-1* *(****or134-1****), Potassium voltage-gated channel subfamily S member 2* *(****kcns2****)* | García-Angulo et al. 2019 | AC278064.1 |
| 31F1 | 53125 | 0,009 | TL11-I | *Probable E3 ubiquitin-protein ligase (****rnf217****)* | García et al. 2019 | AC278109.1 |
| 31N1 | 75632 | 0,012 | TL17-Tl | *Glycogen phosphorylase, muscle form (****pygm****), Neurexin-2 (****nrxn2****)* | García et al. 2019 | AC278050.1 |
| 32B8 | 196621 | 0,032 | STL9-Sc | *Arrestin-C (****arr3****), Probable phospholipid-transporting ATPase IG (****atp11c****), Glycerophosphoinositol inositolphosphodiesterase (****gdpd2****), Phosphatidylinositol 3,4,5-trisphosphate 5-phosphatase 2B (****inppl1b****), Guanine nucleotide exchange factor DBS (****mcf2****), P2Y purinoceptor 4 (****p2ry4****), PDZ domain-containing protein 11 (****pdzd11****), Ras-related protein Rab-6A (****rab6a****), SRY-related HMG-box 3 (****sox3****), PCTP-like protein (****stard10****)* | Portela-Bens et al. 2017 | AC270097.1 |
| 35D17 | 111328 | 0,018 | TL12-Tl | *Sex Determinig Region Y (SRY) box 9 (****sox9****)* | García et al. 2019 | AC278100.1 |
| 36D3 | 65243 | 0,011 | MT1-Tl | *Melanocortin receptor 4 (****mc4r****)* | García-Angulo et al. 2018 | AC278115.1 |
| 36E3 | 43011 | 0,007 | TL15-Pc | *AP-2 complex subunit alpha-2 (****ap2a2****), F-box/LRR-repeat protein 16 (****fbxl16****)* | García et al. 2019 | AC278105.1 |
| 36H2 | 69340 | 0,011 | SMT4-Tl | *Titin (****ttn****), Coiled-coil domain-containing protein 141 (****ccdc141****), Trichohyalin (****tchh****)* | García et al. 2019 | AC278098.1 |
| 36I3 | 34866 | 0,006 | MT2-I | *Chromodomain-helicase-DNA-binding protein 3 (****chd3****), Tumor necrosis factor ligand superfamily member 12 (****tnfsf12****)* | García et al. 2019 | AC278094.1 |
| 36J2 | 56196 | 0,009 | SMT4-Tl | *Gamma-crystallin M3 (****crygm3****)* | García et al. 2019 | AC278110.1 |
| 36K1 | 30968 | 0,005 | MT2-Sc | *Myotubularin-related protein 1 (****mtmr1****)* | García et al. 2019 | AC278080.1 |
| 36M2 | 81656 | 0,013 | TL18-I | *Slit homolog 1 protein (****slit1****)* | García et al. 2019 | AC278090.1 |
| 38F24 | 55286 | 0,009 | TL11-I | *Heat shock protein HSP 90-alpha* *(****hsp90aa1****), Serine/threonine-protein phosphatase 2A 56 kDa* *regulatory subunit* *gamma isoform* *(****ppp2r5c****), Thyroxine 5-deiodinase* *(****dio3****)* | García-Angulo et al. 2020 | AC278114.1 |
| 38N10 | 203362 | 0,033 | MT2-Sc | *Beta-2-microglobulin* *(****b2m****),* *Beta-2-microglobulin****-****like* *(****b2ml****), Protein lifeguard* *(****tmbim4****), Interleukin -1- receptor associated kinase 3* *(****irak3****), Cerebral dopamine neurotrophic factor* *(****cdnf****), Heat shock 70 kDa protein 14* *(****hspa14****), Prolactin* *(****prl****), Neuroepithelial cell-transforming gene 1 protein (****net1****), Ankyrin repeat and SOCS box-containing 13a* *(****asb13a****), Rab GDP dissociation inhibitor beta* *(****gdi2****), Mesencephalic astrocyte-derived neurotrophic factor (****manf****)* | García-Angulo et al. 2019 | AC278095.1 |
| 39F2 | 77120 | 0,013 | STL9-Stl | *Endoplasmic reticulum-Golgi intermediate compartment protein 1* *(****ergic1****), Dual specificity protein phosphatase 1* *(****dusp1****), E3 ubiquitin-protein ligase NEURL1B* *(****neurl1b****), Melatonin receptor type 1C* *(****mel1c****)* | García-Angulo et al. 2020 | AC278072.1 |
| 42P4 | 216432 | 0,035 | TL19-Sc | *Interleukin-20* *(****il20****), Interleukin-10* *(****il10****), MAPK-activated protein kinase 2* *(****mapkapk2****), Dual specificity tyrosine-phosphorylation-regulated kinase 2* *(****dyrk2****), Ras association domain-containing protein 5*  *(****rassf5****), Inhibitor of* *nuclear* *factor kappa-B kinase subunit epsilon* *(****ikbke****), SLIT-ROBO Rho GTPase-activating protein 2* *(****srgap2****), Protein FAM72A* *(****fam72a****)* | García-Angulo et al. 2019 | AC278059.1 |
| 45L11 | 392275^h^ | 0,064 | TL11-I | *Syntaxin-11-like (****stx11****), Monofunctional C1-tetrahydrofolate synthase, mitochondrial (****mthfd1l****), A-kinase anchor protein 12b (****akap12b****), Zinc finger and BTB domain-containing protein 2b (****zbtb2b****), Estrogen receptor alpha (****esr1****), Protein FAM72A (****fam72a****), Ataxin-1-like isoform (****atxn1l****), Bone morphogenetic protein 7-like (****bmp7****), membrane-associated transporter protein (****slc45a2****), Apolipoprotein Db (****apodb****), Rho GTPase-activating protein 21 (****arhgap21****)* | Present work | AC278087.1 |
| 45M23^d^ | 134409 | 0,022 | TL-I | *Proline-rich protein 15-like protein (****prr15l****), Pyridoxine-5'-phosphate oxidase (****pnpo****), 39S ribosomal protein L10, mitochondrial (****mrpl10****), Oxysterol-binding protein-related protein 7 isoform (****osbpl7****), T-box transcription factor TBX21 (****tbx21****), Steroidogenic factor 1 (****nr5a1****),* *Glypican-4-like isoform (****gpc4****), DNA damage-inducible transcript 4-like protein (****ddit4l****)* | Present work | AC278052.1 |
| 46B2 | 175688 | 0,029 | SMT4-Tl | *Multidrug resistance-associated protein 4 (****abcc4****), Claudin-10 (****cldn10****), L-dopachrome tautomerase (****dct****), Transcription factor SOX-21 (****sox21****), DnaJ homolog subfamily C member 3 (****dnjc3****), Glycoprotein glucosyltransferase 2 (****uggt2****), Glypican-6 (****gpc6****)* | García et al. 2019 | AC278074.1 |
| 46C5 | 190621 | 0,031 | MT2-Tl | *Solute carrier family 12 member 3 (****slc12a3****), Myeloid associated differentiation marker homolog (****myadm****), Rho guanine nucleotide exchange factor 11 (****arhgef11****), Heterogeneous nuclear ribonucleoprotein C (****hnrnpc****), FH1/FH2 domain-containing protein (****fhod1****), Kelch-like protein 33 (****klhl33****), Protein mono-ADP-ribosyltransferase PARP14 (****parp14****), Protein NDRG2 (****ndrg2****), Leukotriene B4 receptor 1 (****ltb4r****), Macrophage mannose receptor 1 (****mrc1****), Complement C1q-like protein 3 (****c1ql3****), Preprotein translocase SecA subunit-like protein (****seca****)* | García et al. 2019 | AC278071.1 |
| 46K16^d^ | 274154 | 0,045 | TL-Sc | *NAD(P) transhydrogenase, mitochondrial (****nnt****), lysosome membrane protein 2c (****impc****), fibroblast growth factor 10 (****fgf10****), coiled-coil domain-containing protein 80 (****ccdc80****), ankycorbin (****rai14****), mesoderm-specific transcript homolog protein (****mest****)* | Present work | AC278078.1 |
| 46P22^c^ | 93963 | 0,015 | SMT4-Sc  STL8-Sc | *Thyrotropin-releasing hormone receptor 2* *(****trhr2****), Zinc finger protein, multitype 1 (****zfpm1****)* | García-Angulo et al. 2020 | AC278065.1 |
| 48K7 | 272508^g^ | 0,045 | MT1-Sc  STL6-I | *Doublesex and mab-3-related transcription factor 3 (****dmrt3****), KN motif and ankyrin repeat domain-containing protein 1 (****kank1****), Doublesex and mab-3-related transcription factor 1 (****dmrt1****), Fructose-1,6-bisphosphatase 1 (****fbp1****), Cilia- and flagella-associated protein 157 (****cfap157****), Doublesex and mab-3-related transcription factor 2 (****dmrt2****)* | García-Angulo et al. 2018  Rodríguez et al. 2019 | AC278058.1 |
| 48P7 | 242510 | 0,040 | MT1-I | *7-alpha-hydroxycholest-4-en-3-one 12-alpha-hydroxylase (****cyp8b1****), Parathyroid hormone/parathyroid hormone-related peptide receptor (****pth1r****), Myosin light chain 3 (****myl3****), Corticotropin-releasing factor receptor 2 (****crhr2****), Rho-associated protein kinase 1 (****rock1****), Ubiquitin carboxyl-terminal hydrolase 14 (****usp14****), Aquaporin-1 (****aqp1****), THO complex subunit 1 (****thoc1****)* | García-Angulo et al. 2018  Rodríguez et al. 2019 | AC278048.1 |
| 50K3 | 226363 | 0,037 | TL19-I | *Solute carrier family 16 member 1 (****slc16a1****), Sodium-coupled monocarboxylate transporter 1 (****slc5a8****), Thyrotropin beta (****tshβ****), Mitochondrial glutamate carrier 1 (****slc25a55****), Tetraspanin-2*  *(****tspan2****), Nerve growth factor (****ngf****), Potassium voltage-gated channel subfamily A member 3 (****kcna3****), Potassium voltage-gated channel subfamily A* *member 2 (****kcna2****), Potassium voltage-gated channel subfamily A member 10 (****kcna10****), 60S acidic ribosomal protein* *P2 (****rplp2****), Adenosine receptor A1* *(****adora1****)* | García-Angulo et al. 2020 | AC278047.1 |
| 52C17 | 185039 | 0,030 | MT1-Sc | *Acyl-protein thioesterase 1 (****lypla1****), N-acetyltransferase ESCO1 (****esco1****), Vacuolar protein sorting-associated protein 41 homolog (****vps41****), Transcription factor Sox-17-alpha (****sox17a****), Oxygen-regulated protein 1 (****rp1****), 39S ribosomal protein L15, mitochondrial (****mrpl15****), Regulator of G-protein signaling 20 (****rgs20****), Myosin regulatory light chain 2, smooth muscle (****mlc2****), FAST kinase domain-containing protein 3, mitochondrial (****fastkd3****), Charged multivesicular body protein 5 (****chmp5****), Myomesin-1 (****myom1****), Kappa-type opioid receptor (****oprk1****)* | García-Angulo et al. 2018  Rodríguez et al. 2019 | AC278111.1 |
| 52E17 | 182264 | 0,030 | TL16-Sc | *Ribosomal RNA small subunit methyltransferase NEP1 (****emg1****), C-type natriuretic peptide 1 (****nppc1****), Zyxin (****zyx****), Probable 28S rRNA (cytosine(4447)-C(5))-methyltransferase (****nop2****), Kell blood group glycoprotein (****kel****), Inhibitor of growth protein 4 (****ing4****), Glyceraldehyde-3-phosphate dehydrogenase (****gapdh****), Condensin complex subunit 1 (****ncapd2****), Tumor necrosis factor receptor superfamily member 5 (****cd40****), Tumor necrosis factor receptor superfamily member 1A (****tnfrsf1a****), Retinol-binding protein 1 (****rbp1****), Tapasin-related protein (****tapbpl****), Tyrosine-protein phosphatase non-receptor type 6 (****ptpn6****), Complement C1r subcomponent (****c1r****), Vesicle-associated membrane protein 2 (****vamp2****), Pleckstrin homology domain-containing family G member 6 (****plekhg6****), 39S ribosomal protein L51, mitochondrial (****mrpl51****), Lymphocyte activation 3 protein (****lag3****), Intermediate filament family orphan 1 (****iffo1****)* | García et al. 2019 | MW199162.1 |
| 52G10 | 171874 | 0,028 | MT2-Tl | *Protein-methionine sulfoxide oxidase mical3a (****mical3a****), Protein-methionine sulfoxide oxidase mical3b (****mical3b****), Leukotriene A-4 hydrolase* *(****lta4h****), ADP-ribosylation factor 4* *(****arf4****), Golgi coiled-coil protein 1 (****gcc1****), Protein DENND6B* *(****dennd6b****), V-type proton ATPase subunit F* *(****atp6v1f****), Interferon regulatory factor 5* *(****irf5****), Transportin 3* *(****tpno3****), Short-wave-sensitive opsin 1 (****opn1sw****), Calumenin-A* *(****calua****), Diacylglycerol kinase* *(****dgki****), Cyclic amp responsive element binding protein 3 like* *protein 3 like protein 2* *(****creb3l2****), Single-stranded DNA-binding protein, mitochondrial* *(****ssbp1****), Laminin subunit beta-1 (****lamb1****)* | García-Angulo et al. 2019 | AC278079.1 |
| 53B20 | 1006593 | 0,164 | MT1-Pc | *Leucine-rich repeat-containing protein 32 (****lrrc32****), Myelin protein zero-like protein 1 (****mplz1****), Rab GTPase-binding effector protein 1 (****rabep1****), Vacuolar protein sorting-associated protein 37D (****vps37d****), Extracellular calcium-sensing receptor (****casr****), Probable G-protein coupled receptor 149 (****gpr149****), 1-phosphatidylinositol 4,5-bisphosphate phosphodiesterase eta-1 (****plch1****), G-rich sequence factor 1 (****grsf1****), Protein RUFY3 (****rufy3****), Solute carrier family 4 member 4a (****slc4a4a****), Neuropeptide FF receptor 2a (****npffr2a****), Sn1-specific diacylglycerol lipase beta (****daglb****), C-Jun-amino-terminal kinase-interacting protein 4 (****spag9****), Hemoglobin subunit alpha-D (****hbad****), Hemoglobin subunit beta-1 (****hbb-b1****), Inactive rhomboid protein 1 (****rhbdf1****), Heme transporter hrg1-b (****slc48a1a****), Solute carrier family 26 member 9 (****slc26a9****), Arylalkylamine N-acetyltransferase 2 (****aanat2****), Forkhead box protein O6 (****foxo6****), Poly(rC)-binding protein 4 (****pcbp4****), Protein dispatched homolog 3 (****disp3****), Bone morphogenetic protein 1 (****bmp1****), KAT8 regulatory NSL complex subunit 3 (****kansl3****), Anthrax toxin receptor 1 (****antxr1****), Glutamine--fructose-6-phosphate aminotransferase [isomerizing] 1 (****gfpt1****), NFU1 iron-sulfur cluster scaffold homolog, mitochondrial (****nfu1****), Telomere length and silencing protein 1 (****c9orf78****), Mediator of RNA polymerase II transcription subunit 22 (****med22****), AP2-associated protein kinase 1 (****aak1****), Neprilysin (****mme****)* | Rodríguez et al. 2019 | AC278097.1 |
| 53D20 | 92651 | 0,015 | TL16-Tl | *Antigen peptide transporter 1 (****tap1****), Transcription factor HES-2 (****hes2****), Transcription factor HES-5 (****hes5****), Neurogenic locus notch homolog protein 1 (****notch1****), H-2 class II histocompatibility antigen, E-S beta chain (****h2-eb1****) Vitelline membrane outer layer protein 1 homolog (****vmo1****), Bromodomain-containing protein 2 (****brd2****)* | García-Angulo et al. 2019 | AC278082.1 |
| 53I12^c,d^ | 246103 | 0,040 | TL-Stl  TL-I | *No genes annotated* | Present work | AC278067.1 |
| 54F6^d^ | 563084^j^ | 0,092 | TL-I | *Olfactory receptor 51E2-like (****or51e2****), Beta-arrestin-1 (****arrb1****), Autophagy-related protein 16-2 (****atg16l2****), E3 ubiquitin-protein ligase rififylin (****rffl****), Anoctamin-7 isoform (****ano7****), Somatostatin-1 (****sst1****), Collagen alpha-4(IV) chain-like (****col4a4****), Olfactory receptor 11A1-like (****or11a1****), Vitellogenin Aa (****vtgaa****), Vitellogenin Ab (****vtgab****), Collagen alpha-5(IV) chain (****col4a5****), Mitogen-activated protein kinase-binding protein 1 (****mapkbp1****), Endothelin-converting enzyme 2b (****ece2b****), Eukaryotic translation initiation factor 4H (****eif4h****), Somatostatin-2 (****sst2****)* | Present work | AC278081.1 |
| 55B12 | 44536 | 0,007 | TL21-I | *HAUS augmin-like complex subunit 8* *(****haus8****), SAC3 domain-containing protein 1 (****sac3d1****), C3 and PZP like alpha-2-macroglobulin domain containing 8* *(****cpamd8****), Forkhead box protein E4 (****foxe4****), Forkhead box protein D2* *(****foxd2****)* | García-Angulo et al. 2020 | AC278096.1 |
| 56H24 | 155006 | 0,025 | MT1-C  STL-C  TL-C | *A-kinase anchor protein 9 (****akap9****), Potassium/sodium hyperpolarization-activated cyclic nucleotide-gated channel 4 (****hcn4****), Aquaporin-10 (****aqp10****), HCLS1-associated protein X-1 (****hax1****), Tuftelin (****tuft1****), Ubiquitin-associated protein 2-like (****ubap2l****), Zinc finger protein 687b (****znf687b****), Uncharacterized protein C1orf43 homolog (****c1orf43****), Phosphatidylinositol 4-phosphate 5-kinase type-1 alpha (****pip5k1a****)* | García-Angulo et al. 2018 | AC278049.1 |
| 57G6^d^ | 481179 | 0,079 | TL-Stl | *Immunoglobulin-like domain-containing receptor 1b (****ildr1b****), Immunoglobulin-like domain-containing receptor 2 (****ildr2****), NADP-dependent malic enzyme (****me1****), Four and a half LIM domains protein 3 (****fhl3****), R-spondin-1 (****rspo1****), Galanin receptor 2a (****galr2a****), Shaker-related potassium channel tsha2, Activated CDC42 kinase 1 (****tnk2****), Transferrin receptor 1b (****tfr1b****), Potassium voltage-gated channel subfamily A member 1 (****kcna1****), Bifunctional UDP-N-acetylglucosamine 2-epimerase/N-acetylmannosamine kinase (****gne****), Sodium/potassium/calcium exchanger 2 (****slc24a2****), CCR4-NOT transcription complex subunit 4 (****cnot4****), Sialic acid synthase (****nans****), Importin subunit alpha-8 (****kpna7****), DNA repair protein complementing XP-A cells (****xpa****), Fibroblast growth factor 20 (****fgf20****), Tryptophan 2,3-dioxygenase A (****tdo2****)* | Present work | AC278055.1 |
| 59B23^d^ | 563084^j^ | 0,092 | TL-I | *Vitellogenin Aa (****vtgaa****), vitellogenin Ab (****vtgab****), coiled-coil domain-containing protein 181 (****ccdc181****), transmembrane gamma-carboxyglutamic acid protein 1 (****prrg1****), oxygen-dependent coproporphyrinogen-III oxidase, mitochondrial (****cpox****), prolyl 3-hydroxylase 2 (****p3h2****), TGF-beta-activated kinase 1 and MAP3K7-binding protein 3 (****tab3****), transmembrane protein 47 (****tmem47****), nexilin (****nexn****), afadin- and alpha-actinin-binding protein (****ssx2ip****), di-N-acetylchitobiase (****ctbs****), guanine nucleotide-binding protein G(I)/G(S)/G(O) subunit gamma-5 (****gng5****), spermatogenesis-associated protein 1 (****spata1****), ribosome production factor 1 (****rpf1****), deoxyribonuclease-2-alpha (****dnase2****), Zinc transporter ZIP9-A (****slc39a9-a****), pleckstrin homology domain-containing family D member 1 (****plekhd1****), coiled-coil domain-containing protein 177 (****ccdc177****), beta,beta-carotene 9',10'-oxygenase (****bco2****)* | Present work | AC278075.1 |
| 60P19 | 176179 | 0,029 | MT2-Stl | *FACT complex subunit SPT16* *(****supt16h****), C-type mannose receptor 2 (****mrc2****), Protein FAM234A* *(****fam234a****), Tripartite motif-containing 25* *(****trim25****), Mitochondrial ribosomal protein L48 (****mrpl48****), Astrocytic phosphoprotein PEA-15* *(****pea15****), Calsequestrin* *(****casq1b****), Complement C1q-like protein 2*  *(****c1ql2****), E3 ubiquitin-protein ligase TRIM39 (****trim39****)* | García-Angulo et al. 2020 | AC278093.1 |
| 60P24^d^ | 55742 | 0,009 | TL-I | *SRY-related HMG-box 8 (****sox8****)* | Present work | AC278083.1 |
| 63A3 | 178206 | 0,029 | TL21-Sc | *Eukaryotic translation initiation factor 4 gamma 1 (****eif4g1****), Putative GTP-binding protein 6 (****gtpbp6****), Leukemia NUP98 fusion partner 1 (****lnp1****), Mediator of RNA polymerase II transcription subunit 8 (****med8****), Omega-amidase NIT2 (****nit2****), Pre-B-cell leukemia transcription factor 1 (****pbx1****), PI-PLC X domain-containing protein 1 (****plcxd1****), TBC1 domain family member 23 (****tbc1d23****), Mitochondrial import receptor subunit TOM70 (****tomm70****), ICOS ligand (****icoslg****), Transposable element Tcb1* | García-Cegarra et al. 2013  García et al. 2019 | AC278107.1 |
| 63A7 | 184023 | 0,030 | TL17-Stl | *Protein furry homolog (****fry****), Klotho (****kl****), Neurobeachin (****nbea****), Sister chromatid cohesion protein PDS5 homolog B (****pds5b****), Replication factor C subunit 3 (****rfc3****), STAR-related lipid transfer protein 13 (****stard13****), Zygote arrest protein 1 (****zar1****)* | García-Cegarra et al. 2013 | AC278117.1 |
| 64A8 | 211201 | 0,034 | SMT5-Sc | *Follistatin-A (****fsta****), NADH dehydrogenase [ubiquinone] iron-sulfur protein 4, mitochondrial (****ndufs4****), Integrin alpha-2 (****itga2****), Ankyrin repeat domain 34Bb (****ankrd34bb****), Stromal cell-derived factor 2-like protein 1 (****sdf2l1****)* | Present work | AC278077.1 |
| 65I16 | 22158^i^ | 0,004 | TL17-I | *protein turtle homolog B (****igsf9b****), myb/SANT-like DNA-binding domain-containing protein 2 (****msantd2****)* | Present work | AC278119.1 |
| 68G4 | 248974 | 0,041 | TL10-Sc | *Tektin-2 (****tekt2****), ETS domain-containing transcription factor ERF- like (****erfl****), protein capicua homolog (****cic****), free fatty acid receptor 3 (****ffar3****), upstream stimulatory factor 2-like (****usf2****), lamin-A (****lmna****), protein argonaute 3 (****ago3****), glycogen synthase kinase-3 beta (****gsk3b****)* | Present work | AC278099.1 |
| 71J13^d^ | 257109 | 0,042 | TL-Stl | *Transcription factor Sp5-like (****sp5l****), Ankyrin repeat domain-containing protein 33B-like (****ankrd33b****), Glycoprotein-N-acetylgalactosamine 3-beta-galactosyltransferase 1-A (****c1galt1a****), Tachykinin-3 (****tac3****), Zinc finger protein 385A (****znf385a****), Histone-lysine N-methyltransferase 2D isoform (****kmt2d****), Solute carrier family 26 member 10 (****slc26a10****), Beta-1,4 N-acetylgalactosaminyltransferase 1-like (****b4galnt1****), Galectin-8-like isoform (****lgals8****), Ribonucleoside-diphosphate reductase subunit M2-like (****rrm2****), Activin receptor type-1B (****acvr1b****), Serine/threonine-protein kinase receptor R3 (****acvrl1****), Rho guanine nucleotide exchange factor 25 (****arhgef25****)* | Present work | AC278103.1 |
| 71N11 | 172136 | 0,028 | TL16-Tl | *Apoptosis-associated speck-like protein containing a CARD* *(****pycard****), Sodium channel subunit beta-1* *(****scn1b****), Tumor necrosis factor* *(****tnf****), SH3-binding domain protein 5* *(****sh3bp5****), Alpha-1,3-mannosyl-glycoprotein-2-beta-N-acetylglucosaminyltransferase (****mgat1****), Ring finger protein 183* *(****rnf183****), DEAH-box helicase 16* *(****dhx16****), Lysosomal thioesterase PPT2 (****ppt2****), Friend leukemia integration 1 (****fli1****), ETS domain-containing protein* *(****etv2****), DnaJ homolog subfamily C member 28* *(****dnajc28****)* | García-Angulo et al. 2019 | AC278056.1 |
| 73B7 | 704705^e^ | 0,115 | MT1-Stl | *Rho GTPase-activating protein 21 (****arhgap21****), Apolipoprotein D (****apod****), Otospiralin (****otos****)* | García-Angulo et al. 2018  Rodríguez et al. 2019 | AC278063.1 |
| 73J17^d^ | 284596 | 0,046 | TL-Sc | *Androgen receptor (****ar****), Leucine-rich repeat and immunoglobulin-like domain-containing nogo receptor-interacting protein 2 (****lingo2****), Centrosomal protein of 164 kDa (****cep164****), Nuclear receptor coactivator 7 (****ncoa7****)* | Present work | AC278069.1 |

^a^ Referred to a 612,3 Mb genome size of *Solea senegalensis*, as described by Manchado et al. (2019).

^b^ Total length of 7 BAC clones that were taken together (3F15, 9E8, 13E1, 19K18, 19L16, 22C2 and 25P16).

^c^ BAC clone with multiple FISH signals in which it was not possible to identify the main signal and, therefore, the homologue region.

^d^ BAC clone in which it could not identify the specific chromosome hybridization.

^e^ Total length considering the overlapping among 5K5, 10K23, 10L10 and 73B7 BAC clones.

^f^ Total length considering the overlapping between 11O20 and 20D18 BAC clones.

^g^ Total length considering the overlapping between 16E16 and 48K7 BAC clones.

^h^ Total length considering the overlapping between 45L11 and 4E10 BAC clones.

^i^ BAC size calculated considering the overlapping region with the 19K18 BAC clone.

^j^ Total length considering the overlapping between 54F6 and 59B23 BAC clones.
