# Supplementary material for: A Comprehensive Integrated Genetic Map of the Complete Karyotype of Solea senegalensis (Kaup 1858)
Source: Genes (Basel). 2020 Dec 31;12(1):49. doi: 10.3390/genes12010049 (PMC7824234; doi:10.3390/genes12010049)
Supplement: Supplementary file 1 [file genes-12-00049-s001.zip › Table S2.docx]

**Table S2.** Group of conserved linkages across the different species treated in the study and the GO link of each gene.

| ***S. senegalensis* chromosome** | **Gene** | **UniProt database link to Gene Ontology** |
| --- | --- | --- |
| 1 | *cyp8b1* | Q9UNU6 |
|  | *rock1* | Q13464 |
|  | *usp14* | P54578 |
|  | *aqp1* | P29972 |
|  | *thoc1* | Q96FV9 |
|  | *crhr2* | Q13324 |
|  | *myl3* | P08590 |
|  | *pth1r* | Q03431 |
|  | *klf2* | Q9Y5W3 |
|  | *eps15l1* | Q9UBC2 |
|  | *rx2* | Q9I9A2 |
|  | *calr* | P27797 |
|  | *tpm1* | P13104* |
|  | *tpm4* | P67936 |
|  | *rab8a* | P61006 |
|  | *cib3* | Q96Q77 |
|  | *ap1m1* | A0A0R4IRS1* |
|  | *ankrd45* | No results |
|  | *tmem70* | Q9BUB7 |
| 4 | *adat3* | Q8JFW4* |
|  | *pms1* | P54277 |
|  | *fkbp7* | Q9Y680 |
|  | *mstn* | O42222* |
|  | *hibch* | Q58EB4* |
|  | *gls* | O94925 |
|  | *osgepl1* | Q32LQ3* |
|  | *akap* | No results |
|  | *ormdl1* | Q8JFB7 |
|  | *tlr7* | Q9NYK1 |
|  | *tlr8* | Q9NR97 |
|  | *mid1* | O15344 |
|  | *cog3* | Q96JB2 |
|  | *frmpd4* | Q14CM0 |
|  | *pcdh8* | O95206 |
|  | *egfl6* | Q8IUX8 |
|  | *vangl1* | Q8TAA9 |
|  | *dopey2* | Q9Y3R5 |
|  | *morc3a* | Q14149 |
|  | *slc5a3* | P53794 |
|  | *casq2a* | O14958 |
|  | *ptprn* | Q16849 |
|  | *kcne2* | Q9Y6J6 |
|  | *rpe* | A9C3R8* |
|  | *igsf3* | O75054 |
|  | *nhlh2* | Q02577 |
|  | *ackr3* | P25106 |
|  | *crygm3* | Q5XTM9* |
|  | *ttn* | Q8WZ42 |
|  | *ccdc141* | No results |
|  | *tchh* | Q07283 |
|  | *pde11a* | Q9HCR9 |
|  | *osbpl6* | Q9BZF3 |
| 16 | *tap1* | Q03518 |
|  | *notch1* | P46530* |
|  | *brd2* | P25440 |
|  | *pycard* | Q9I9N6* |
|  | *scn1b* | Q07699 |
|  | *fli1* | Q01543 |
|  | *ets1b* | P14921 |
|  | *emg1* | Q92979 |
|  | *nppc1* | P23582 |
|  | *iffo1* | No results |
|  | *zyx* | Q15942 |
|  | *nop2* | P46087 |
|  | *kel* | P23276 |
|  | *gapdh* | Q5XJ10* |
|  | *cd40* | P29965 |
|  | *tnfrsf1a* | P19438 |
|  | *rbp5* | P82980 |
|  | *tapbpl* | Q9BX59 |
|  | *ptpn6* | P29350 |
|  | *c1r* | P00736 |
|  | *vamp2* | P63045 |
|  | *plekhg6* | Q3KR16 |
|  | *mrpl51* | Q5BJJ8* |

* GO links of fish species.
